# Supplementary material for: Modeling glioblastoma heterogeneity as a dynamic network of cell states
Source: Mol Syst Biol. 2021 Sep 16;17(9):e10105. doi: 10.15252/msb.202010105 (PMC8444284; doi:10.15252/msb.202010105)
Supplement: Supplementary file 6 — Source Data for Figure 5 [file MSB-17-e10105-s004.zip › Figure5A_sourcedata/GSEA_3071/nosigdb_stateIII.GseaPreranked.1622795155562/DOROSZKO_U3013MG_CLUSTER_0.html]

Details for gene set DOROSZKO\_U3013MG\_CLUSTER\_0[GSEA]

|  || Dataset | state33071 |
| Phenotype | NoPhenotypeAvailable |
| Upregulated in class | na\_neg |
| GeneSet | DOROSZKO\_U3013MG\_CLUSTER\_0 |
| Enrichment Score (ES) | -0.78071856 |
| Normalized Enrichment Score (NES) | -3.1464956 |
| Nominal p-value | 0.0 |
| FDR q-value | 0.0 |
| FWER p-Value | 0.0 |
Table: GSEA Results Summary

  

Fig 1: Enrichment plot: DOROSZKO\_U3013MG\_CLUSTER\_0      
 Profile of the Running ES Score & Positions of GeneSet Members on the Rank Ordered List

  

| PROBE | GENE SYMBOL | GENE\_TITLE | RANK IN GENE LIST | RANK METRIC SCORE | RUNNING ES | CORE ENRICHMENT || 1 | BCHE |  |  | 867 | -0.272 | -0.2019 | No |
| 2 | KLF4 |  |  | 973 | -0.278 | -0.2085 | No |
| 3 | PGM2L1 |  |  | 2260 | -0.354 | -0.5116 | No |
| 4 | TCIM |  |  | 3095 | -0.430 | -0.6935 | No |
| 5 | PCSK1N |  |  | 3346 | -0.470 | -0.7232 | No |
| 6 | CKB |  |  | 3572 | -0.536 | -0.7417 | Yes |
| 7 | NES |  |  | 3596 | -0.545 | -0.7079 | Yes |
| 8 | MAP2 |  |  | 3640 | -0.572 | -0.6773 | Yes |
| 9 | VCAN |  |  | 3741 | -0.660 | -0.6548 | Yes |
| 10 | SYT11 |  |  | 3748 | -0.669 | -0.6077 | Yes |
| 11 | F3 |  |  | 3764 | -0.693 | -0.5611 | Yes |
| 12 | NRXN2 |  |  | 3841 | -0.814 | -0.5213 | Yes |
| 13 | CAVIN2 |  |  | 3860 | -0.862 | -0.4632 | Yes |
| 14 | COL6A1 |  |  | 3863 | -0.872 | -0.4003 | Yes |
| 15 | NGFR |  |  | 3872 | -0.937 | -0.3342 | Yes |
| 16 | TNC |  |  | 3874 | -0.951 | -0.2652 | Yes |
| 17 | IGFBP5 |  |  | 3895 | -1.082 | -0.1916 | Yes |
| 18 | PTPRZ1 |  |  | 3913 | -1.342 | -0.0983 | Yes |
| 19 | IGFBP2 |  |  | 3917 | -1.404 | 0.0031 | Yes |
Table: GSEA details [plain text format]

  

Fig 2: DOROSZKO\_U3013MG\_CLUSTER\_0: Random ES distribution      
 Gene set null distribution of ES for **DOROSZKO\_U3013MG\_CLUSTER\_0**
